# Supplementary material for: A Cross-Sectional Study on Physical Activity and Psychological Distress in Adults with Asthma
Source: Healthcare (Basel). 2022 Dec 7;10(12):2469. doi: 10.3390/healthcare10122469 (PMC9778577; doi:10.3390/healthcare10122469)
Supplement: Supplementary file 1 [file healthcare-10-02469-s001.zip › Table S1. Self-perceived health, according to physical activity level in Spanish adults with asthma.pdf]

Table S1. Self-perceived health, according to physical activity level in Spanish adults with asthma.

	Physical Activity Levels											
Variables	Inactive		Walkers		Active		VeryActive					
SPH	n (%)		n (%)		n (%)		n (%)		x^2	df	p	CC
Negative	90a	59.6%	227b	47.0%	86c	30.4%	29c	23.6%	57.0	3	<0.001	0.228
Positive	61a	40.4%	256b	53.0%	197c	69.6%	94c	76.4%				

n (participants); % (percentage); x2 (Pearson's Chi-square); df (Degree freedom); p (p-value); CC (Contigency coefficient).
